# Eukaryotic Microbial Communities in Japanese Arable Andisols Investigated by Amplicon Sequencing of 18S rRNA Genes

**DOI:** 10.1128/MRA.00218-20

**Published:** 2020-06-04

**Authors:** Kazumori Mise, Shigeto Otsuka

**Affiliations:** a Department of Applied Biological Chemistry, Graduate School of Agricultural and Life Sciences, the University of Tokyo, Tokyo, Japan; b Department of Biological Sciences, Graduate School of Science, the University of Tokyo, Tokyo, Japan; c Collaborative Research Institute for Innovative Microbiology, the University of Tokyo, Tokyo, Japan; University of California, Riverside

## Abstract

Compared with the well-studied soil prokaryotic communities, little is known about soil eukaryotic communities. Here, we investigated the eukaryotic community structures in 43 arable soils using amplicon sequencing of 18S rRNA genes. Major taxonomic groups, such as Fungi, Holozoa, and Stramenopiles, were detected in all samples.

## ANNOUNCEMENT

Recent developments in high-throughput sequencing have unveiled soil microbial community structures in detail. While numerous studies have reported prokaryotic community structures in soil (1), much less is known about eukaryotic communities. Nevertheless, eukaryotic microbes play vital roles in the soil ecosystem; they drive biogeochemical cycling (2), prey on prokaryotes (3), and are potential plant pathogens (4). To fill this knowledge gap, we investigated eukaryotic community structures in Japanese arable soils by using well-established 18S rRNA gene amplicon sequencing.

Details on the soil sampling have previously been reported (5). Briefly, 43 arable Andisols sampled from 11 regions in Japan (32.9° to 43.0°, 130.7° to 143.3°) were investigated. For each region, soil was sampled from one to six (different) cropland field(s). They included both fallow (*n* = 7) and planted soils (*n* = 36), with various crop rotations and fertilization regimes. Collected soil samples were sieved through 2-mm mesh and stored at −30°C. DNA was extracted from 400 to 500 mg of soil using a FastDNA spin kit for soil (Qbiogene, Carlsbad, CA, USA) following the manufacturer’s protocol, with a modification that a casein solution (2% [wt/vol]; pH adjusted to 8.0 with 300 mM sodium phosphate buffer) was added to mitigate DNA adsorption to soil minerals (6). The partial sequences of 18S rRNA genes, typically 100 to 150 bases long, were amplified using the fusion primers Euk1391f and EukBr (7, 8) that incorporated index and Illumina adaptor sequences. PCR conditions included an initial 3 minutes of denaturation at 72°C, followed by 25 cycles of denaturation (94°C, 30 s), annealing (57°C, 45 s), and extension (72°C, 1 min). The amplicons were purified by electrophoresis in agarose gels with subsequent reextraction from the gel using the Wizard SV gel and PCR cleanup system (Promega, Madison, WI, USA). The amplicon libraries from all samples were mixed in equimolar amounts and sequenced on a MiSeq instrument (Illumina, CA, USA), generating 150-bp paired-end reads. Paired-end reads with >20 bases of overlapping regions were merged, and low-quality sequences with expected errors of 0.5 bases or more were eliminated using USEARCH v11.0.667 (9). In cases where one read goes beyond the beginning (5′ end) of the opposite read, the exceeded regions were trimmed. Furthermore, merged sequences falling beyond the range of 100 to 150 bases long (8) were discarded. Sequences passing these filtering steps were taxonomically annotated using naive Bayesian classifier (NBC) implemented in QIIME v1.9.1 (10, 11), trained with SILVA v132 (12), and optimized for NBC (https://www.arb-silva.de/fileadmin/silva_databases/qiime/Silva_132_release.zip), with a bootstrap confidence value threshold of 0.5. Potential plant-derived sequences (i.e., sequences annotated as Chloroplastida) and unannotated sequences were discarded.

**FIG 1:**
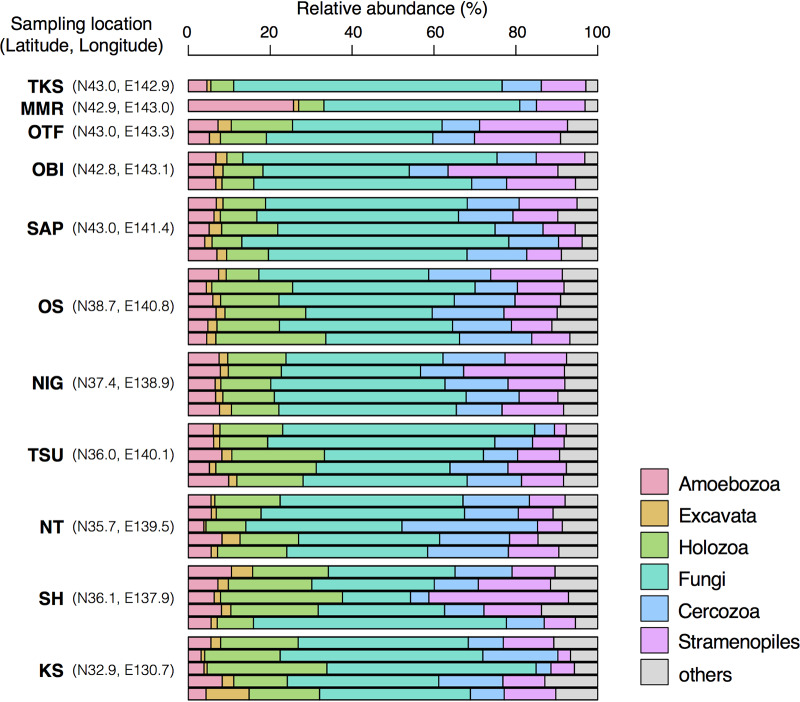
Community structures of eukaryotic microbes in 43 Japanese arable Andisols based on 18S rRNA gene sequencing. Sampling locations of the sequenced soil samples are indicated on the left. Detailed information on each sample is available in a previous report (5). Abbreviations: TKS, Tokachi-Shimizu; MMR, Memuro; OTF, Otofuke; OBI, Obihiro; SAP, Sapporo; OS, Osaki; NIG, Niigata; TSU, Tsukuba; NT, Nishi-Tokyo; SH, Shiojiri; KS, Koshi.

Within the total 2,053,072 high-quality sequences, 1,595,038 (77.7%) were successfully annotated (37,093 sequences per sample on average). Dominant taxonomic clades included Fungi (43.3% ± 10.6%, mean ± SD), Holozoa (14.7% ± 6.13%), Stramenopiles (12.4% ± 6.13%), Cercozoa (12.4% ± 5.06%), Amoebozoa (6.71% ± 3.36%), and Excavata (2.20% ± 1.57%) (Fig. 1).

### Data availability.

The amplicon sequence data have been deposited in DDBJ/ENA/GenBank under the accession number PRJDB6544 and also under DRA009572.
